# Biotechnology clusters needs innovative minds and critical mass

**DOI:** 10.1186/1753-6561-8-S4-O41

**Published:** 2014-10-01

**Authors:** Marcos Pinotti, Evgeniya Shamis

**Affiliations:** 1 Universidade Federal de Minas Gerais, Belo Horizonte, Minas Gerais, Brazil

## 

The development of biotechnology industry is strongly based on the interaction with research laboratories and universities. The process of creating new companies and bringing new products to the market depend on how the actors are prepared and aware about the innovation process. Successful movements towards acceleration startups have overcome two major difficulties: Dissemination of the innovation virus in the academy and get enough critical mass of brain and resources. How long does it take to change the order of magnitude of the production of a new material from milligrams to tons? A PhD student probably has speculated about the final use of the new biomaterial introduced subcutaneously in some (un)fortunate mice. It is important to make researchers aware that one does not need to leave the comfort zone and face risks in a jungle to think big and find a noble application for their pet biomaterial. It is known that creative energy may be used beyond the Petri's dishes, bioreactors, scientific papers and patent applications. The ideas, like stem cells, may differentiate in whatever product the scientist mind would imagine. To play the game of innovation, scientists must learn new rules and new metrics. To be successful in this race, one must work hard and have a strategy. Probably, the podium will not be achieved in the first race but the sweat drops will evaporate from the road as quickly as the entrepreneur is getting better. He/she will evolve. So as patents and papers will evolve to business plan, in vitro/in vivo test will evolve to clinical trials or to medical prescriptions and inventions will transform into innovations. In order to survive in the 21st Century environment, the new species of entrepreneur with PhD degree, Homo innovatus, will populate biotechnology startups. In more evolved ecosystems, ideas and knowledge are translated into innovation. The process becomes easier when there is a minimum of critical mass of people and companies. The combination of quality, diversity and large number of ideas makes huge difference in a cluster. The main challenge is how to concentrate bright people in one place. There are a number of strategies to make it happen. The process of attracting talents to a region is to provide infrastructure for good life quality, i.e., high quality elementary and secondary schools for the children, nice hospitals, variety of cultural and touristic attractions. It is also necessary to create a flow of bright people into the place. The best way of achieving this is to have a number of universities that provide high quality workforce for the companies. The reputation of good salaries, good place to raise the family, good cultural activities help immensely to create and strengthen the brand of the cluster. Other than traditional metrics, the impact of cluster on the local and regional economic development and quality of life will be decisive on the success of attraction of talents and investments to a city or region.
